# The “long tail” of the protein tumbling correlation function: observation by ^1^H NMR relaxometry in a wide frequency and concentration range

**DOI:** 10.1007/s10858-015-0001-1

**Published:** 2015-11-18

**Authors:** Matthias Roos, Marius Hofmann, Susanne Link, Maria Ott, Jochen Balbach, Ernst Rössler, Kay Saalwächter, Alexey Krushelnitsky

**Affiliations:** Institut für Physik, Martin-Luther-Universität Halle-Wittenberg, Betty-Heimann-Str. 7, 06120 Halle, Germany; Universität Bayreuth, Lehrstuhl Experimentalphysik II, Universitätsstr. 30, 95440 Bayreuth, Germany

**Keywords:** Inter-protein interactions, Brownian tumbling, Field-cycling, Relaxation, Correlation function

## Abstract

Inter-protein interactions in solution affect the auto-correlation function of Brownian tumbling not only in terms of a simple increase of the correlation time, they also lead to the appearance of a weak slow component (“long tail”) of the correlation function due to a slowly changing local anisotropy of the microenvironment. The conventional protocol of correlation time estimation from the relaxation rate ratio *R*_1_/*R*_2_ assumes a single-component tumbling correlation function, and thus can provide incorrect results as soon as the “long tail” is of relevance. This effect, however, has been underestimated in many instances. In this work we present a detailed systematic study of the tumbling correlation function of two proteins, lysozyme and bovine serum albumin, at different concentrations and temperatures using proton field-cycling relaxometry combined with *R*_1ρ_ and *R*_2_ measurements. Unlike high-field NMR relaxation methods, these techniques enable a detailed study of dynamics on a time scale longer than the normal protein tumbling correlation time and, thus, a reliable estimate of the parameters of the “long tail”. In this work we analyze the concentration dependence of the intensity and correlation time of the slow component and perform simulations of high-field ^15^N NMR relaxation data demonstrating the importance of taking the “long tail” in the analysis into account.

**Electronic supplementary material:**

The online version of this article (doi:10.1007/s10858-015-0001-1) contains supplementary material, which is available to authorized users.

## Introduction

Overall Brownian tumbling of proteins in solution is an important issue in many biophysical and biochemical studies, and may provide information on the size and shape of the protein under investigation, as well as on inter-molecular interactions. Starting from the pioneering works by Kay et al. (1989) and Clore et al. (1990), a long series of papers has been published that deal with high-resolution NMR relaxation studies of internal dynamics of proteins in solution. Almost all these studies utilized the well known model-free approach (Lipari and Szabo 1982a, b) for relaxation times analysis. According to the simplest form of this approach, the normalized second-order reorientational correlation function reads

1 $$ C(t) = \exp ( - t/\uptau_{\text{rot}} )\left[ {S_{\text{int}}^{2} + \left( {1 - S_{\text{int}}^{2} } \right)\exp ( - t/\uptau_{\text{int}} )} \right], $$

where τ_rot_ is the correlation time of the overall protein Brownian rotation (tumbling), and $S_{\text{int}}^{2}$ and τ_int_ are the order parameter and the correlation time of the internal motion, respectively. More sophisticated protocols take into account different components of the internal mobility, the non-spherical shape of the protein molecule leading to anisotropic overall motion and the contribution of chemical exchange to the relaxation rate *R*_2_, as surveyed in a number of reviews (Daragan and Mayo 1997; Korzhnev et al. 2001; Palmer 2001; Boehr et al. 2006; Kleckner and Foster 2011; Ishima 2012; Saito 2014). The main target of those studies was providing site-specific information on internal motions. However, as seen from Eq. (1), correct estimation of the internal dynamics parameters ($S_{\text{int}}^{2}$ and τ_int_) is impossible without an exact determination of the molecule’s overall rotational correlation time τ_rot_. To a first approximation τ_rot_ can be obtained from the ratio of the spin–lattice and spin–spin relaxation rates, *R*_1_/*R*_2_, measured for the most rigid residues of a protein undergoing (almost) no slow internal mobility (Kay et al. 1989; Clore et al. 1990). Then, τ_rot_ is usually refined during the global fit of all the data. This approach assumes a free rotation of the molecule to be described in terms of a single exponential, or at most a few exponentials describing the non-spherical shape of a protein molecule. In our opinion, this treatment can lead to imprecise results, in particular at high protein concentrations.

**Fig. 1:**
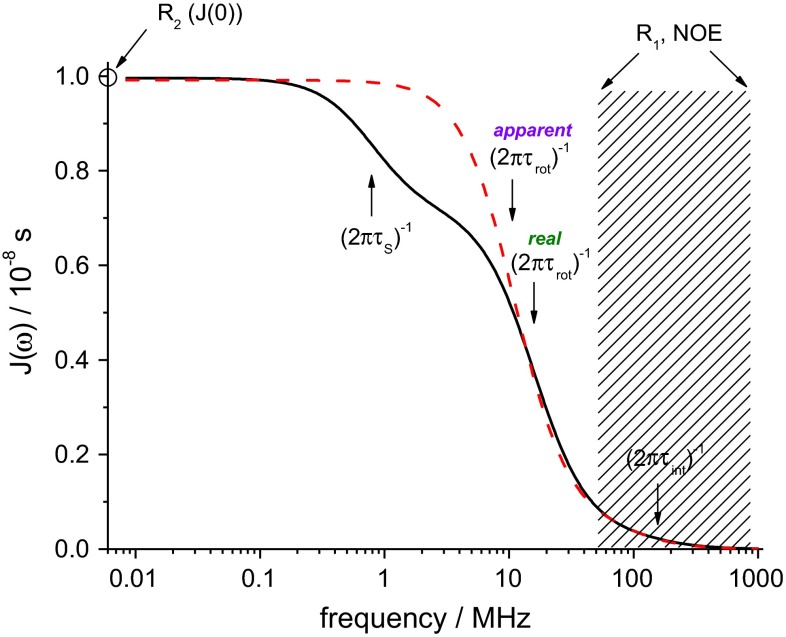
Representation of the spectral density function as directly sampled by relaxation parameters for the example of ^15^N(–^1^H) relaxation. Three dispersions are relevant, corresponding to three modes of motion, see Eq. (2); the inflection points of the dispersions corresponding to the condition ωτ = 1 are marked by *arrows*. The hatched area marks the frequency range sampled by *R*_1_’s and *NOE*’s measured at the ^1^H resonance frequencies from 500 to 800 MHz. *R*_2_ provides the value of the low-frequency limit of the spectral density. The frequencies in-between are not accessible by high-field relaxation measurements. The “long tail” dispersion is located right in this gap. The *dashed line* indicates the “apparent” spectral density as obtained from the relaxation data neglecting the impact of the “long tail”. The “real” and the “apparent” spectral densities were simulated according to the dynamic parameters presented for the “mobile” residue, Fig. 7 at $S_{\text{rot}}^{2}$τ_S_ = 4 ns (see details in the final part of the paper)

In a series of preceding papers (Krushelnitsky and Fedotov 1993; Ermolina et al. 1993; Ermakova et al. 2002; Krushelnitsky 2006; Roos et al. 2015) it has been shown that inter-protein long-range electrostatic interactions not just increase τ_rot_, but cause the appearance of a weak slowly decaying component of the tumbling correlation function (“long tail”). At protein concentrations of a few mM (as typical for protein NMR samples) the inter-protein distances are comparable with protein’s size, and the energy of mutual electrostatic steering is comparable with the thermal energy *kT* (Ermolina et al. 1993). The microsurrounding around each protein induces a local anisotropy of the “normal” Brownian tumbling. The lifetime of this local anisotropic configuration of proteins is controlled by micro-environmental fluctuations primarily mediated by the translational motion of proteins in respect to each other, rendering this lifetime considerably longer than τ_rot_. Thus, the correlation function (1) can be better approximated by (Krushelnitsky 2006)

2 $$ C(t) = \exp ( - t/\uptau_{\text{S}} )\left[ {S_{\text{rot}}^{2} + \left( {1 - S_{\text{rot}}^{2} } \right)\exp ( - t/\uptau_{\text{rot}} )} \right] \cdot \left[ {S_{\text{int}}^{2} + \left( {1 - S_{\text{int}}^{2} } \right)\exp ( - t/\uptau_{\text{int}} )} \right], $$

where $S_{\text{rot}}^{2}$ is the order parameter of the local Brownian rotation anisotropy and τ_S_ is the slow correlation time characterizing the lifetime of this anisotropy. Note that on a long time scale, protein rotation remains fully isotropic, and $S_{\text{rot}}^{2}$ characterizes the Brownian rotation anisotropy solely on a time scale of τ_rot_. As long as the protein concentration is not very high, $S_{\text{rot}}^{2}$ is usually very small, i.e. values around 1 % or even less. At first glance, this practically negligible component should have no significant effect on the relaxation rates. However, one has to keep in mind that the relaxation rates are proportional to frequency-dependent values of the spectral density *J*(ω), which is the Fourier transform of *C*(*t*). The effect of the “long tail” on relaxation rates is demonstrated in Fig. 1, where it is depicted that high-field relaxation measurements are not affected by the slow component. In contrast, *R*_2_ is proportional to the spectral density function at zero frequency *J*(0), or equivalently, to the time integral over the correlation function $\int\limits_{0}^{\infty } {C\left( t \right)} \,{\text{d}}t$:

3 $$ J(0) \sim \left( {1 - S_{\text{rot}}^{ 2} } \right)\uptau_{\text{rot}} + S_{\text{rot}}^{ 2} \uptau_{\text{S}} . $$

Since τ_rot_ ≪ τ_S_, the two terms in Eq. (3) may be comparable to each other in spite of $S_{\text{rot}}^{2}$ ≪ 1. Thus, *R*_2_ is appreciably affected by the “long tail” of the tumbling correlation function, such that the standard protocol of high-field NMR relaxation rates analysis may produce imprecise results when applied to protein solutions that are not highly diluted, see Fig. 1.

The “long tail” can hardly be recognized in the analysis of a conventional set of high-field relaxation parameters (*R*_1_, *R*_2_ and *NOE* measured at several resonance frequencies, usually from 500 to 900 MHz for protons), meaning that these data can always be well fitted assuming the standard approach, as we demonstrate below. In fact, the slow component can hardly be seen in the analysis since these measurements do not sample the low frequency range of *J*(ω), as clearly shown in Fig. 1. When, however, much lower frequencies in high resolution ^15^N relaxation experiments are sampled using a field-shuttling system, the existence of the additional contribution to *R*_2_ rates becomes evident for practically all residues in ubiquitin, where it likewise was admitted that this effect can hardly be explained by chemical exchange (Charlier et al. 2013). It is very likely that this contribution to *R*_2_’s also results from the “long tail”. It is worth noting that the relaxation measurements at resonance frequencies of several MHz (as was also done in our previous papers mentioned above) can detect the slow component, but cannot provide the parameters $S_{\text{rot}}^{2}$ and τ_S_, separately: from Eq. (3) it follows that only the product $S_{\text{rot}}^{2}$ τ_S_ can be safely obtained from the analysis of these data.

Although the existence of the “long tail” is by now well documented, detailed data characterizing its behavior at different conditions are very sparse and incomplete due to the methodological challenges described above. To obtain exact quantitative information on the “long tail”, one has to measure the relaxation times at low resonance frequencies (~1 MHz and below), which is only possible using field-cycling (FC) NMR relaxometry (Koenig and Brown 1990; Kimmich and Anoardo 2004; Kruk et al. 2012). However, almost all FC experiments in protein solutions by now dealt with the solvent (H_2_O or D_2_O) instead of the protein signal, as FC NMR features a low sensitivity. The analysis of the water spin–lattice relaxation rate dispersions $R_{1} \left( \omega \right)$ can indeed resolve the “long tail” of the protein tumbling correlation function (Krushelnitsky and Fedotov 1993; Krushelnitsky 2006), yet these estimates can be affected by the finite lifetime of water molecules within the protein structure (Denisov and Halle 1996). If the water lifetime overlaps with τ_S_, the parameters of the slow component cannot be determined accurately.

Measuring the protein signal in the FC experiments is more challenging, and we are aware of very few applications of this kind so far which benefitted from the increased sensitivity of modern instruments (Bertini et al. 2005; Luchinat and Parigi 2007). The key point of these important studies was to demonstrate that the integral proton signal can be used to extract an average order parameter that serves as a faithful global measure of internal protein flexibility. The data shown do exhibit indications of the “long tail” (Bertini et al. 2005), but the related gradual increase of *R*_1_ at low frequencies was discussed in terms of protein aggregation. As discussed earlier (Krushelnitsky 2006), protein aggregation is an unlikely explanation for the appearance of the slow component in the tumbling correlation function. The results shown below will provide additional evidence that protein aggregation can hardly explain the slow component, at least not in the general case.

In this work we conduct a systematic study of the “long tail” of the tumbling correlation function using ^1^H FC NMR relaxometry of protein protons in D_2_O solutions combined with standard *R*_2_ and *R*_1ρ_ measurements that complement the FC NMR data at low frequencies. The main questions targeted in this study are: how do the parameters of the “long tail” behave upon varying the protein concentration?; how critical is the neglect of the “long tail” in the high-field relaxation data analysis?; how small should the protein concentration be for safely neglecting the “long tail”? We try to answer these questions by means of relaxation times analysis, simulations, and considering independent literature data.

## Materials and methods

### NMR experiments

The frequency dependence (dispersion) of the spin–lattice relaxation rate $R_{1} \left( \omega \right)$ was measured with a commercially available Stelar FC 2000 relaxometer located at the University of Bayreuth. It allows for proton frequencies of $2\uppi \cdot 10\,{\text{kHz}} \le \upomega = \upgamma_{H} B_{0} \le 2\uppi \cdot 20\,{\text{MHz,}}$ with $\gamma_{H}$ denoting the gyromagnetic ratio and $B_{0}$ the magnetic field, respectively. The latter is generated by an electromagnet and thus variable. The typical time necessary for switching and stabilizing the coil current is around 2 ms. Basics of electronic FC NMR are discussed extensively in the literature (Kimmich and Anoardo 2004). The temperature can be varied within $- 120\,^{\circ} {\text{C}} \le T \le 180\,{^\circ } {\text{C}}$ while for the present contribution only a small interval of $4\,{^\circ } {\text{C}} \le T \le 48\,{^\circ } {\text{C}}$ could be covered avoiding protein freezing and denaturation of the sample. In order to decompose the magnetization decay into protein and residual water (HDO, H_2_O) contributions the polarization time was always 3 s, enough to provide sufficient water signal. The analysis of the multi-component magnetization decays will be discussed below. Although the relaxometer enables measurements at the frequencies down to 10 kHz, in our FC experiments the lowest frequency was only 100 kHz. At lower frequencies the relaxation rates could only be determined with some uncertainty, especially at high protein concentrations, see below. Details on this issue are presented in the “Appendix”. This limitation, however, was not significant since the resulting frequency gap is complemented by the *R*_1ρ_ measurements.

*R*_2_ and *R*_1_ at 20 MHz were measured using a Bruker Minispec mq20. The low resonance frequency for *R*_2_ experiments allows avoiding the contribution of chemical exchange of protein protons to *R*_2_ (Luz and Meiboom 1963; Hills et al. 1989). *R*_1ρ_ measurements were preformed on a Bruker Avance II spectrometer with a magnetic field corresponding to 400 MHz proton Larmor frequency. Since we used spin-lock fields of 20 and 40 kHz, the contribution of chemical exchange to the *R*_1ρ_ relaxation rates is likewise negligible. *R*_2_, *R*_1_ at 20 MHz and *R*_1ρ_ were measured in Halle on the same samples as in Bayreuth.

Prior to all measurements the protein-D_2_O solutions of different concentrations were filled into thoroughly cleaned NMR glass tubes. We abstained from degassing the samples to avoid D_2_O evaporation and altered concentrations. For all experiments, we applied single short-pulse excitation with a sufficiently large spectral width of at least 50 kHz to ensure that all protein protons contribute equally to the integral signal. The accuracy of the temperature calibration and stabilization was in all cases better than ±1 °C.

### Sample preparation

Lysozyme (LYZ), M_W_ = 14,300, from chicken egg white and fatty acid free bovine serum albumin (BSA), M_W_ = 66,500, were delivered from Sigma-Aldrich (product numbers 62970 and A7030, respectively) as lyophilized powders. Both proteins were dissolved in D_2_O for few hours and lyophilized again to maximally remove labile protons that would increase the residual water signal. Afterwards, the protein was dissolved in D_2_O again without adding salt or buffer. The pH obtained was pH 3.8 for LYZ and pH 7.0 for BSA and is well distinguished from the isoelectric point (pH 11.35 and pH 4.7, respectively). No significant changes in the pH (more than 0.1–0.2) were observed upon varying the protein concentration.

**Fig. 2:**
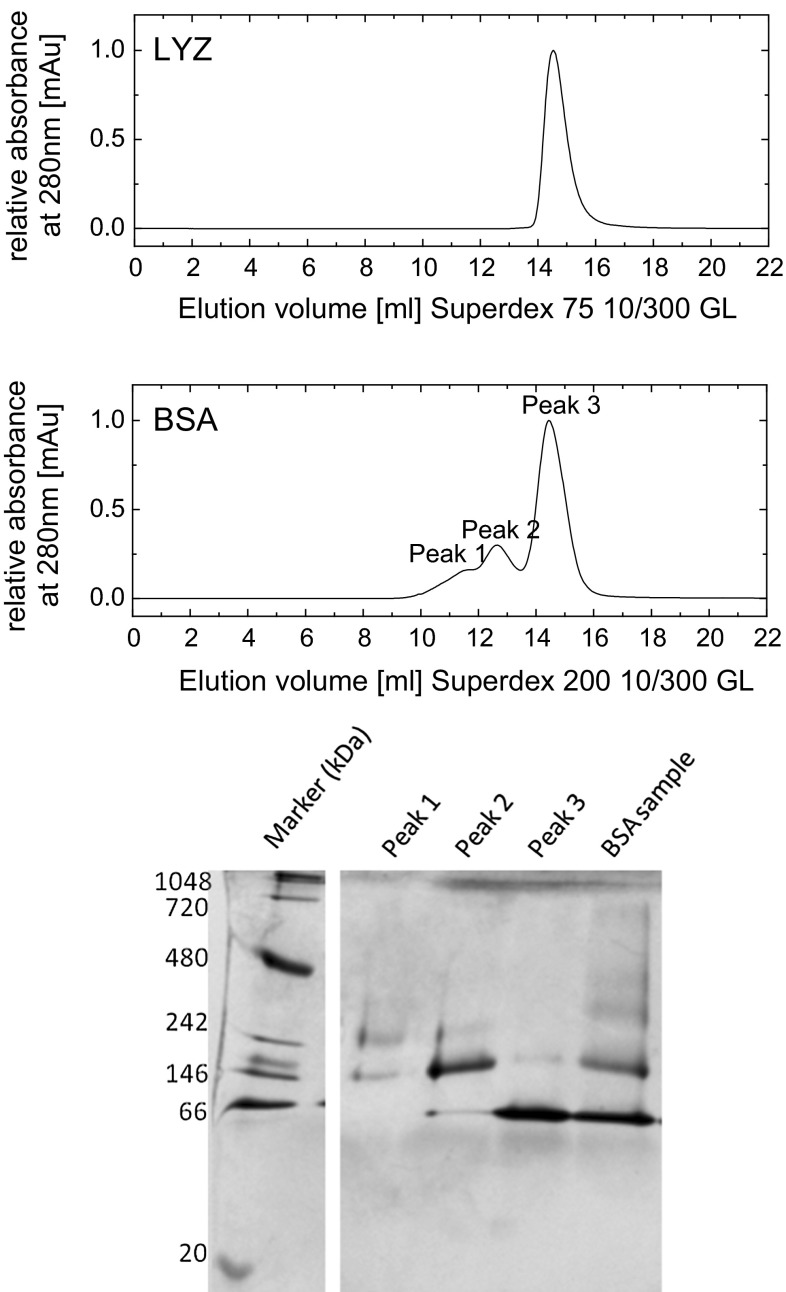
Size-exclusion chromatogram of LYZ and BSA (*top*) and blue native (BN) PAGE of BSA performed on the elution volume of different peaks of the size-exclusion chromatography (*bottom*)

Size-exclusion chromatography at a flow rate of 0.4 ml/min and blue native polyacrylamide electrophoresis (BN-PAGE) reveal the monodispersity of the LYZ sample and the polydispersity of the BSA sample (Fig. 2). The BSA polydispersity corresponds well to previous observations (Squire et al. 1968; Atmeh et al. 2007). To avoid unspecific interaction of the proteins with the column material, the elution buffer is adjusted to 50 mM NaCl in the case of BSA and 50 mM Na-phosphate buffer, 50 mM NaCl at pH 7.5 in the case of LYZ. The BN-PAGE of BSA was performed based on the method of Schägger et al. (1994) using a native unstained protein marker obtained from life technologies.

In our experiments we observed that at temperatures above 28–30 °C BSA solutions reveal a slow (on the time scale of several hours) increase of the oligomers portion. For this reason, for all BSA samples we limited the temperature range of the experiments to $4\,{^\circ } {\text{C}} \le T \le 26\,{^\circ } {\text{C}}{.}$

## Results and discussion

### Analysis of the relaxation decays

**Fig. 3:**
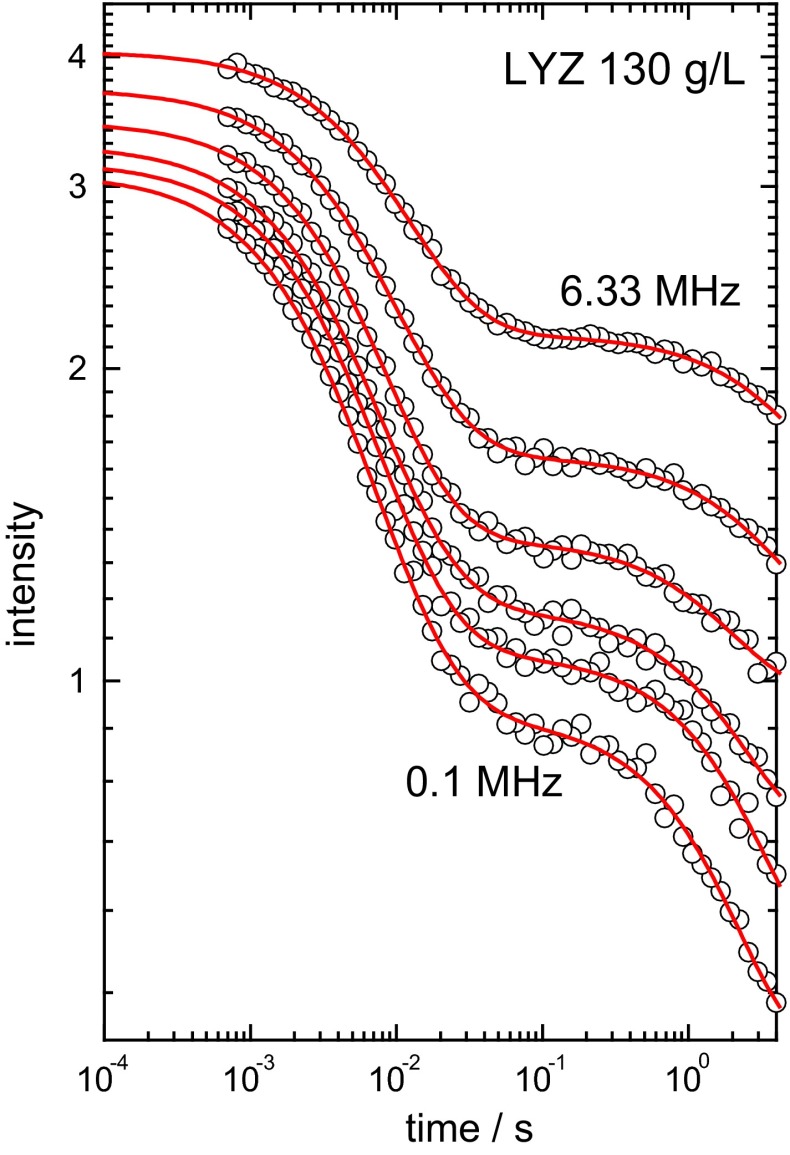
FC-NMR relaxation decays of 130 mg/ml LYZ at selected relaxation field strengths (from *top to bottom*): 6.33, 3.77, 2.25, 1.34, 0.795, 0.1 MHz. *Solid lines* are the log-normal fits combined with a single-exponential decay of the residual water protons (Eq. 4)

**Fig. 4:**
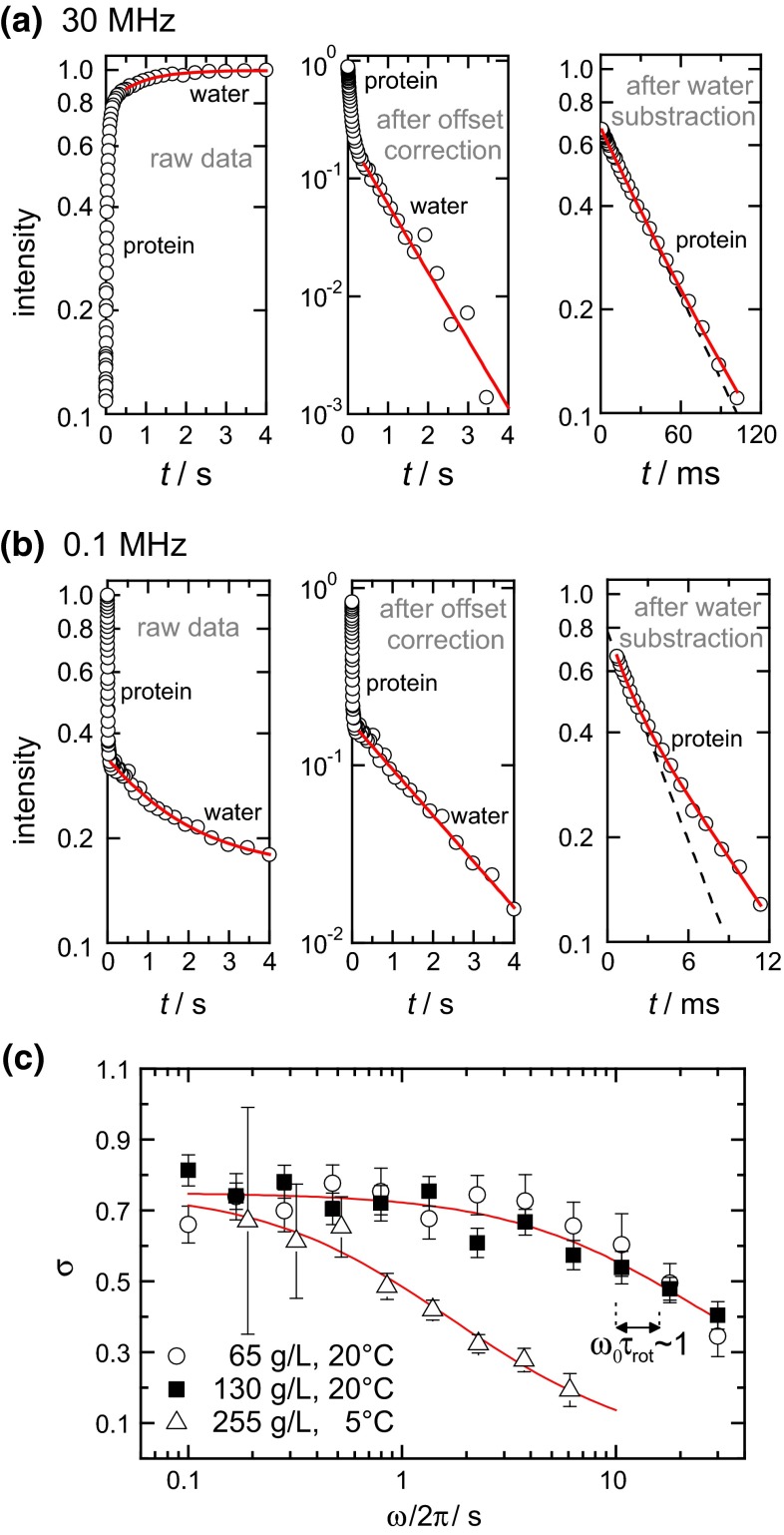
Relaxation decays in a 130 mg/ml lysozme sample at (**a**) 30 MHz and (**b**) 0.1 MHz and separate fitting result (*solid lines*) of the water and the protein signal. *Left* (**a**, **b**) Intensities versus relaxation delay as directly obtained in the field-cycling experiment. *Middle* (**a**, **b**): After subtracting the value of the equilibrium magnetization, the mono-exponential decay of the water protons can be clearly seen. *Right* (**a**, **b**): Protein signal as observed after subtracting the water signal. *Dotted lines* indicate the initial slope of the decays, i.e. the mean relaxation rate. (**c**) Distribution width parameter of LYZ *R*_1_s for three concentrations as a function of the proton resonance frequency as obtained by a log-normal fit, see Eq. 4. *Vertical dotted lines* indicate the frequencies obeying the condition ω_0_τ = 1 for the protein concentrations 65 and 130 mg/ml. *Red solid lines* are polynomial fits to guide an eye

Figures 3 and 4 present examples of the FC-NMR relaxation decays of the same sample at different magnetic field strengths. The relaxation decays consist of two components: the fast and slow decaying components belong to protein and residual water protons, respectively (Krushelnitsky and Fedotov 1993). Figure 3 shows raw data and Fig. 4 demonstrates the procedure of the water component subtraction and the form of the pure protein component at two different resonance frequencies [similar plots for the transversal magnetization decays are shown in ref. (Roos et al. 2015)]. Because of the fast exchange between hydrated and bulk water molecules (Denisov and Halle 1996), the water component is always single-exponential, whereas the protein component is not (Krushelnitsky 2006; Luchinat and Parigi 2007; Roos et al. 2015).

The raw decays were fitted using a sum of the water and the protein components, the latter featuring a log-normal distribution of the relaxation rates:

4 $$ \begin{aligned} I(t) & = \left( {I_{ 0} - I_{\text{eq}} } \right)\,f_{\text{RLX}} (t) + I_{\text{eq}} \\ f_{\text{RLX}} (t) & = \left( {1 - p_{\text{W}} } \right)\int\limits_{0}^{\infty } {p(R_{1} )\,\exp \left( { - R_{1} \,t} \right)dR_{1} } + p_{\text{W}} \exp \left( { - R_{\text{W}} \,t} \right) \\ p(R_{1} ) & = \frac{1}{{\sqrt {2\pi \sigma^{2} } R_{1} }}\exp \left[ {\frac{{ - \left( {\ln [R_{1} ] - \ln [R_{\text{median}} ]} \right)^{2} }}{{2\sigma^{2} }}} \right], \\ \end{aligned} $$

where *I*_0_ and *I*_eq_ correspond to the initial and equilibrium intensity, respectively, $p_{\text{W}}$ and *R*_W_ are the relative amplitude and relaxation rate of residual water protons, respectively. $R_{\text{median}}$ and σ correspond to the most populated relaxation rate ($p(R_{\text{median}} )$ has a maximal value) and the distribution width parameter (0 < $\sigma$ < ∞; 0 corresponds to the delta-function, infinity means infinitely wide distribution) of the protein component, respectively. The integral in Eq. (4) was calculated numerically during the fitting. The mean (arithmetic average) of the protein relaxation rates

5 $$ \left\langle {R_{1} } \right\rangle = \int\limits_{0}^{\infty } {p(R_{1} )\,R_{1} \,{\text{d}}R_{1} } = R_{\text{median}} \cdot \exp \left( {\frac{{\sigma^{2} }}{2}} \right) $$

equals the initial slope of the relaxation decay and is proportional to the mean spectral density function $\left\langle {J(\upomega )} \right\rangle$ (that is the spectral density function averaged over all protein protons), as explained in the Supporting Information of ref. (Krushelnitsky et al. 2014). For the subsequent analysis, we use the mean relaxation rate values defined by Eq. (5) for all types of experiments, that is *R*_1_, *R*_1ρ_ and *R*_2_. The dispersion profiles of the water component were not analyzed because of the inferior signal-to-noise ratio. The water component intensity in all samples was several times less than that of the protein; in addition, the long-time plateau limit of the relaxation decays was not always measured because of the long delays. Increasing the quality of these data would require significant increase of the measuring time, which was practically not affordable.

Defining the mean relaxation rates using the log-normal distribution and previously used (Roos et al. 2015) bi-exponential decomposition of the protein component provides in most cases essentially the same results, see Electronic Supporting Material (ESM), Fig. S2. However, the log-normal distribution enables a quantitative characterization of the non-exponentiality of the protein proton relaxation component. Figure 4c shows the distribution width parameter σ as a function of the resonance frequency. For 65 and 130 mg/ml, this dependence corresponds well to the theoretical prediction (Kalk and Berendsen 1976): if ωτ ≫ 1 (τ is the correlation time of the protein Brownian tumbling), the rate of the spin exchange between protons in a protein is much faster than the relaxation rate of individual protons, rendering the integral relaxation decay singly exponential. Yet, even at relatively high frequencies (ω > 10 MHz) the inequality ωτ ≫ 1 does not hold strictly, and thus the decay is still somewhat non-exponential. On the opposite, at ωτ ≪ 1 spin exchange is slow in comparison to spin relaxation. Thus, the intrinsic distribution width of longitudinal relaxation times is observed. At 255 mg/ml at low temperatures, in turn, the anisotropy of Brownian tumbling (“long tail”) is quite pronounced, and the effective correlation time of Brownian tumbling becomes much longer. Spin exchange becomes much more efficient, so that the distribution width σ is reduced to lower values even at lower frequencies. A similar behavior of the shape of the relaxation decay as a function of frequency in FC experiments was reported earlier by Luchinat and Parigi (2007).

We stress that neglecting the distribution of longitudinal relaxation times and fitting such decay with a single exponent may provide quantitatively incorrect results. It is worth mentioning that, irrespective of whether spin exchange is fast or slow, the arithmetic average relaxation rate (as defined by the initial slope) is not affected by spin exchange (Kalk and Berendsen 1976). To avoid possible misunderstandings, we stress that the non-exponential form of relaxation decays bears absolutely no relation to a potentially non-exponential correlation function of motion.

**Fig. 5:**
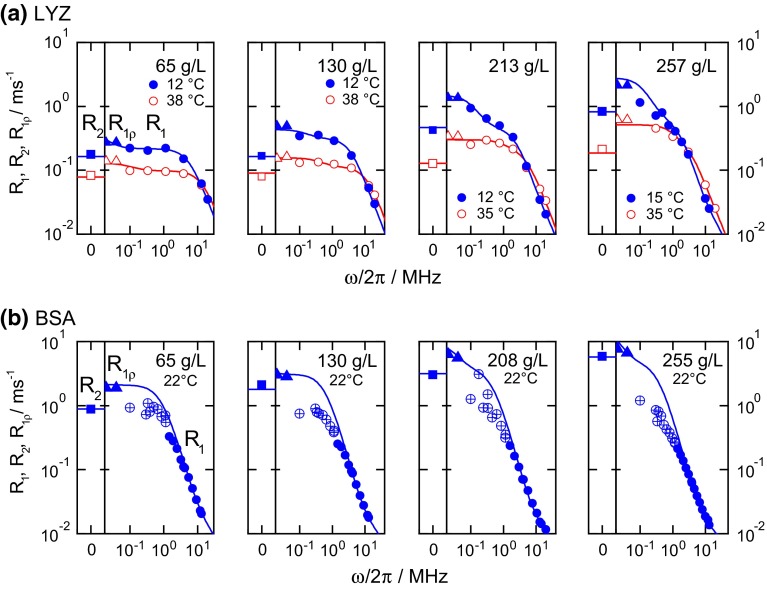
Dispersion profiles of (**a**) LYZ and (**b**) BSA at different concentrations. For direct visual comparison of *R*_1ρ_ (*triangles*) and *R*_1_ (*circles*), *R*_1ρ_ data were multiplied by 10/3 (see ESM, Eqs. S1–S5). *R*_2_’s (*squares*) were measured at 20 MHz and are shown in a separate column of each plot. *Solid lines* provide the best fit result. Uncertain data points (BSA at ω_0_/2π < 3 MHz, see text) were not taken into account for fitting (*crossed symbols*). For BSA, a detailed frequency dependence was recorded only at 22 °C; at other temperatures the data for only few frequencies were measured. The here shown fit to the BSA data assumes monomers only. A fit result involving oligomers is presented in the ESM, Fig. S4. For both proteins, the data shown here were fitted together with all the available data shown in ESM, Figs. S3 and S5

Figure 5 presents exemplary *R*_1_ dispersions for LYZ and BSA along with the fitting curves (see below for the fitting procedure) for different concentrations (the full set of the dispersion data are presented in ESM, Figs. S3–S5). The low-frequency FC relaxation data for BSA solutions are unfortunately not suitable for the analysis, which has two reasons. The first one is the fact that the switching time in FC-experiments (switching from polarization field down to the relaxation field and then back to the detection field) is comparable to the relaxation time. In the “Appendix” we show that this induces no error for a single-exponential decay, but may lead to erroneous results in the case of multi-exponential relaxation decays. The presence of oligomers in the BSA solution (see “Materials and methods” section) causes an additional source of distribution of the *R*_1_ relaxation rates, which leads to a much larger deviation from a mono-exponential behavior of the BSA relaxation decays at low frequencies in comparison with LYZ.

The second reason is the shortest possible relaxation delay in the FC-experiments which was 0.7 ms (“dead time”). Because of the large τ_rot_, the relaxation time of BSA protons at low frequencies is quite short and it is comparable to this “dead time”. The remaining long-time decay appears to be close to singly exponential, but the mean rate (initial slope) is severely underestimated, as demonstrated by Fig. S1. As a criterion whether the respective measurement is reliable or not, the initial slope as provided from the log-normal fit (Eq. 5) was compared to the initial slope as obtained by the bi-exponential decomposition, see ESM, Fig. S2. If and only if these two were the same, the data were considered as being reliable for the analysis. For LYZ, in turn, the spread of relaxation times within one decay curve is not only less pronounced, but also the FC relaxation times are longer, rendering the issue of ill-defined initial slopes less problematic. In fact, for LYZ at all frequencies measured, the initial slope defined from bi-exponential decomposition matches within the experimental error the initial slope of the log-normal fits (Fig. S2).

Another way of presenting the data, namely, temperature dependencies of relaxation rates at several fixed frequencies, is shown in Fig. S5. If *R*_1_ is plotted against inverse temperature 1/*T*, the slope of the data already provides information whether ωτ_rot_ < 1 (positive slope) or ωτ_rot_ > 1 (negative slope). For this reason, the temperature dependencies of the relaxation times measured at various frequencies enable much more precise fitting and more confident identification of different motional processes in comparison with frequency dependencies only.

### Analysis of the relaxation times

The *R*_2_, *R*_1ρ_ and *R*_1_ relaxation times of protein protons are dominated by the homonuclear dipole–dipole relaxation mechanism,

6 $$ R_{1} = \frac{2}{3}K_{HH} \left( {J(\upomega_{0} ) + 4J(2\upomega_{0} )} \right) $$

7 $$ R_{1\rho } = \frac{1}{3}K_{HH} \left( {3J(2\upomega_{1} ) + 5J(\upomega_{0} ) + 2J(2\upomega_{0} )} \right) $$

8 $$ R_{2} = \frac{1}{3}K_{HH} \left( {3J(0) + 5J(\upomega_{0} ) + 2J(2\upomega_{0} )} \right), $$

where *K*_HH_ is the mean dipole–dipole coupling of protons in the protein, *J*(ω) is the spectral density function, and ω_0_/2π and ω_1_/2π are the proton resonance and spin-lock frequencies, respectively. Assuming that τ_int_ ≪ τ_rot_ ≪ τ_S_, the spectral density function can be derived from Eq. (2) as

9 $$ J(\omega ) = \left( {1 - S_{\text{int}}^{2} } \right)\frac{{\tau_{\text{int}} }}{{1 + \left( {\omega \tau_{\text{int}} } \right)^{2} }} + S_{\text{int}}^{2} \left( {1 - S_{\text{rot}}^{2} } \right)\frac{{\tau_{\text{rot}} }}{{1 + \left( {\omega \tau_{\text{rot}} } \right)^{2} }} + S_{\text{int}}^{2} S_{\text{rot}}^{2} \frac{{\tau_{\text{S}} }}{{1 + \left( {\omega \tau_{\text{S}} } \right)^{2} }}. $$

Since we analyzed relaxation times measured at different temperatures, we assumed an Arrhenius temperature dependence for the motional correlation times:

10 $$ \tau_{\text{S,rot,int}} = \tau_{\text{S,rot,int}} (293K)\exp \left[ {\frac{{E_{\text{S,rot,int}} }}{R}\left( {\frac{1}{T} - \frac{1}{293K}} \right)} \right]. $$

Note that in reality the temperature dependencies of the correlation times may deviate from the Arrhenius law, however, within the relatively narrow temperature range of our experiments this approximation works quite well. Overall, the set of fitting parameters included the correlation times τ_int_, τ_rot_ and τ_S_ at 20 °C, the activation energies *E*_int_, *E*_rot_ and *E*_S_, the order parameters $S_{\text{int}}^{2}$ and $S_{\text{rot}}^{2} ,$ and the mean proton coupling constant *K*_HH_. Although the existence of the “long tail” was established in our previous papers, we demonstrate here again that neglecting it in the fitting model leads to systematic inconsistency between the experimental and fitting values of the relaxation rates, see ESM, Fig. S6.

The number of the fitting parameters is quite large, yet the number of experimental data was much larger (overall for all temperatures and concentrations, LYZ: 361 data points, BSA: 262 data points) and, thus, the fitting was quite stable and provides reasonable fitting uncertainties. The values of the fitting parameters were obtained from the simultaneous (global) Monte-Carlo fit of the set of all relaxation times measured at all temperatures and concentrations. The fitting aimed to minimize the root mean square deviation

11 $$ RMSD = \sqrt {\frac{1}{N}\sum\limits_{i = 1}^{N} {\left( {1 - \frac{{R_{\exp } }}{{R_{\text{sim}} }}} \right)}^{2} }, $$

where *R*_exp_ and *R*_sim_ are the simulated (according to the current set of the fitting parameters) and experimental relaxation times, respectively, and *N* is the number of all relaxation times. While fitting, the parameters of the internal motions ($S_{\text{int}}^{2}$, *E*_int_ and τ_int_) as well as *K*_HH_ were assumed to be the same for all concentrations (that is why we fitted the data at all concentrations at a time); all other parameters were assumed to be concentration-dependent. Tables S1 and S2 of ESM contain all the fitting parameters for the two proteins at all concentrations investigated.

A special remark on internal motions is in order. The internal motion parameters are not of central interest in this study, yet they need to be included in the analysis since relaxation times at high frequencies (around 10 MHz and higher) have an appreciable contribution from the first term in Eq. (9), and neglecting it may lead to an incorrect estimation of other parameters. We describe internal motions by a single correlation time τ_int_, however, we actually assume two components of the internal motions since the mean proton coupling constant *K*_HH_ (Eqs. 6–8) is kept as a freely adjustable fitting parameter. Hence, fast internal motions contribute to the *K*_HH_ motional averaging, so that the parameters $S_{\text{int}}^{2}$ and τ_int_ only reflect the slow mode of internal dynamics. Indeed, the rigid lattice value of *K*_HH_ for globular proteins is around 1.3 × 10^10^ s^−2^. Fast methyl proton rotation reduces *K*_HH_ down to ~0.85 × 10^10^ s^−2^ (Krushelnitsky et al. 1996), whereas the *K*_HH_ fitting value (see Tables S1 and S2) is around 0.6 × 10^10^ s^−2^. For a more accurate evaluation of the internal motion parameters $S_{\text{int}}^{2}$ and τ_int_ (and thus more precise estimation of the overall tumbling parameters), we included previously published data on ^1^H *R*_1_ temperature dependencies in BSA and LYZ solutions measured at 11, 27 and 90 MHz (Krushelnitsky 2006) (*R*_2_ data from this work were not included). The relaxation decays in ref. (Krushelnitsky 2006) were analyzed according to the same protocol as in this study, thus the relaxation times can be compared directly. These *R*_1_ data were added to the set of relaxation times at the lowest concentration (65 mg/ml).

The fact that the apparent internal correlation times were in all cases a factor of at least 5–25 shorter than the global tumbling times (see Tables S1 and S2) demonstrates that slower internal motions of some residues, reaching the timescale of global rotation and thus potentially distorting the tumbling correlation function at its τ_rot_-related onset, are probably sparse. If they were abundant, we would expect the separation to be less clear.

For LYZ, the fitting provides a rotational correlation time τ_rot_(*T* = 20 °C) = (10.5 ± 0.2) ns at the lowest concentration measured in this study. We also estimated τ_rot_ using the Stokes–Einstein–Debye law and the values of the viscosity of the protein solution (1.538 ± 0.010 mPa s, measured by the micro-viscometer mVROC, Rheosense, San Ramon, CA) and lysozyme’s hydrodynamic radius of 1.9 nm (Parmar and Muschol 2009). The result appeared to be (10.9 ± 0.1) ns, which is in a good accordance with our analysis.

The analysis of the BSA data is less unambiguous since BSA solutions contain a significant portion of oligomers, see “Materials and methods” section. We applied a more complicated form of the rotational correlation function that contains an additional component attributed to oligomers, see ESM, Eq. S8. However, this more sophisticated analysis does not change significantly the behavior of the “long tail” parameters. Note, however, that τ_rot_ for BSA monomers obtained from this fitting (40 ns) matches well the independent literature value (Ferrer et al. 2001) confirming the adequacy of this analysis in spite of the increased number of fitting parameters.

### Concentration dependence of the “long tail” parameters

**Fig. 6:**
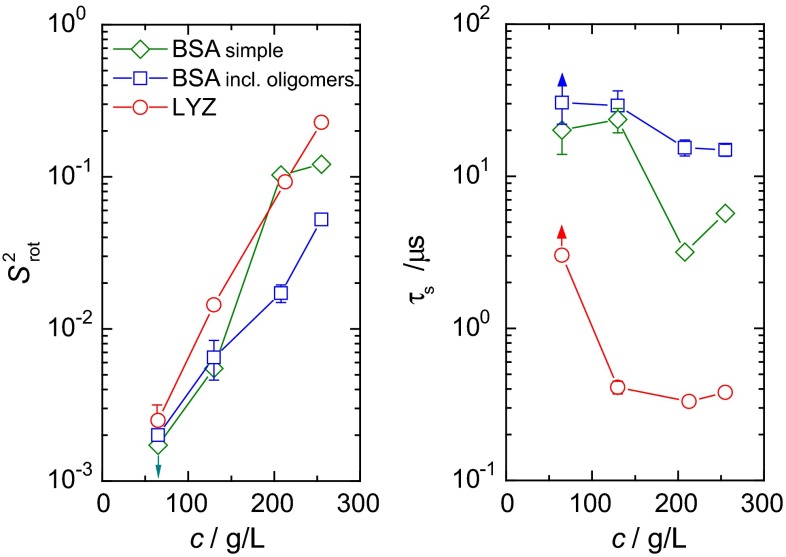
Order parameter $S_{\text{rot}}^{2}$ and correlation time of the slow component $\tau_{\text{S}}$(T = 20 °C) of rotational diffusion as a function of protein concentration of LYZ (*circles*) and BSA (*squares and diamonds*). For BSA, two sets of data are shown corresponding to the analyses using the simple and the more complicated form of the correlation function that includes oligomers (see ESM, Table S2). At the lowest concentration (65 g/L), the upper and lower boundaries of $S_{\text{rot}}^{2}$ and $\tau_{\text{S}} ,$ respectively, are shown (indicated by *arrows*). The minimum (65 g/L) and maximum (260 g/L) concentrations correspond to 4.5 and 18.2 mM for LYZ and 1 and 3.9 mM for BSA, respectively

In our previous studies (Krushelnitsky and Fedotov 1993; Krushelnitsky 2006; Roos et al. 2015) we could not estimate the parameters of the slow component, $S_{\text{rot}}^{2}$ and τ_S_, separately for the reasons described in the Introduction. Combining FC NMR and routine *R*_2_ and *R*_1ρ_ measurements, we now can overcome this issue, and the concentration dependence of $S_{\text{rot}}^{2}$ and τ_S_ can be resolved. Figure 6 presents the concentration dependence of these two parameters for both proteins. At lowest concentration, however, we still cannot determine reliably the parameters $S_{\text{rot}}^{2}$ and τ_S_ separately, since τ_S_ appears to be too long and the dispersion step corresponding to the “long tail” (Fig. 1) falls into a “dead zone” between *R*_2_ and the *R*_1ρ_’s. Consequently, for this concentration, Fig. 6 shows merely high and low limits for $S_{\text{rot}}^{2}$ and τ_S_, respectively, indicated by arrows. At higher concentrations, τ_S_ is shorter (see below), and both “long tail” parameters could be obtained from the fitting independently.

The behavior of $S_{\text{rot}}^{2}$ appears reasonable: with increasing concentration, the average distance between proteins shortens, resulting in stronger inter-protein interactions. This increases the anisotropy of local Brownian rotation and, hence, the order parameter $S_{\text{rot}}^{2}$ becomes larger.

In contrast, τ_S_ decreases with increasing concentration, which seems unexpected. In our view, this tendency can be explained as follows. The lifetime of the local anisotropy relies on the time span needed to change the local environment of the particle (i.e. the protein). With decreasing inter-protein distances (i.e. increasing concentration), fluctuations in the particle alignment have an increased impact on the actual configuration of the particle’s local environment. The lifetime of a certain particle configuration is in particular limited by translational diffusion on the length scale of neighboring particles. Therefore, τ_S_ becomes shorter with increasing protein concentration, at least until a certain concentration close to jamming conditions is reached. Such a trend can also be seen in collective diffusion coefficients measured by dynamic light scattering (Heinen et al. 2012); yet the detailed concentration dependence looks different due to the different natures and time scales of the two processes.

At the same time, we have to admit that this simple explanation cannot explain the τ_S_ concentration dependence quantitatively, especially the significant decrease of τ_S_ between the first and the second concentration points for LYZ. In part, such behavior may be caused by experimental uncertainty since only few relaxation rates report on τ_S_ and $S_{\text{rot}}^{2}$ at low concentrations, where $S_{\text{rot}}^{2}$ is very small. More importantly, any simplified consideration of the slow tail of rotational diffusion does not take into account multi-body interactions, as well as real sizes and shapes of the proteins and their charge distributions. All these aspects render the physics very complex, and simplified models can be expected to account for the qualitative behavior only. We believe that the quantitative description of the “long tail” parameters is only possible using Brownian dynamics simulations that take into account the real shape and the sophisticated electrostatic structure of the protein of consideration (McGuffee and Elcock 2006).

The observed concentration dependence of τ_S_ supports the previous conclusion on the protein aggregation as an unlikely source of the “long tail” (Krushelnitsky 2006). A small portion of large aggregates would also lead to appearance of the slow component; this, however, would be physically unrealistic: large aggregates without an appreciable amount of small oligomers do not conform to Ostwald’s dilution law. The concentration dependence of τ_S_ for both proteins indicates that the size of potential oligomers must decrease with increasing concentration, which is neither physical nor conceivable (see for comparison the behavior of the correlation time of the oligomer component in the BSA data analysis, Table S2, part B). Moreover, if large stable aggregates were present in protein solution, one would observe two components in the magnetization decays with distinctly different relaxation times corresponding to monomers and aggregates, which is not the case. Thus, one may conclude that the protein aggregation does not contribute, at least significantly, to the “long tail”.

### Influence of neglecting the “long tail” while fitting high-field NMR relaxation data

For high-field NMR relaxation studies of protein dynamics the important question is: what is the effect of neglecting the “long tail” in the routine model-free analysis of the relaxation data? To check this aspect, we simulated and then fitted the ^15^N *R*_1_, *R*_2_ and *NOE* data at three proton resonance frequencies—500, 600 and 800 MHz. Overall, we analyzed a set of nine experimental (simulated) parameters. The relaxation parameters *R*_1_, *R*_2_ and *NOE* were simulated assuming the correlation function to be in the form of Eq. (2), see ESM, Fig. S7. In all cases we assumed τ_rot_ = 10 ns, and simulated the data for two cases: a “rigid” residue ($S_{\text{int}}^{2}$ = 0.95, *τ*_int_ = 50 ps) and a “mobile” one ($S_{\text{int}}^{2}$ = 0.7, τ_int_ = 1 ns). These values of the internal motion parameters are typical for residues in the secondary structure elements and (partially) unstructured domains, respectively. For both cases, we simulated the relaxation data assuming four different amplitudes of the slow component: $S_{\text{rot}}^{2}$ = 0, 0.005, 0.01, and 0.02, where *τ*_S_ was fixed to 200 ns in all cases (As mentioned above, only the product $S_{\text{rot}}^{2}$ · *τ*_S_ matters for the analysis). It should be mentioned that the $S_{\text{rot}}^{2}$ values derived from the ^1^H non-selective relaxometry cannot be directly transferred into the analysis of ^15^N relaxation data since different N–H vectors may experience different rotational anisotropies depending on their orientation within the protein structure. Still, the order of magnitude of $S_{\text{rot}}^{2}$ for ^1^H and ^15^N relaxation should be the same.

**Fig. 7:**
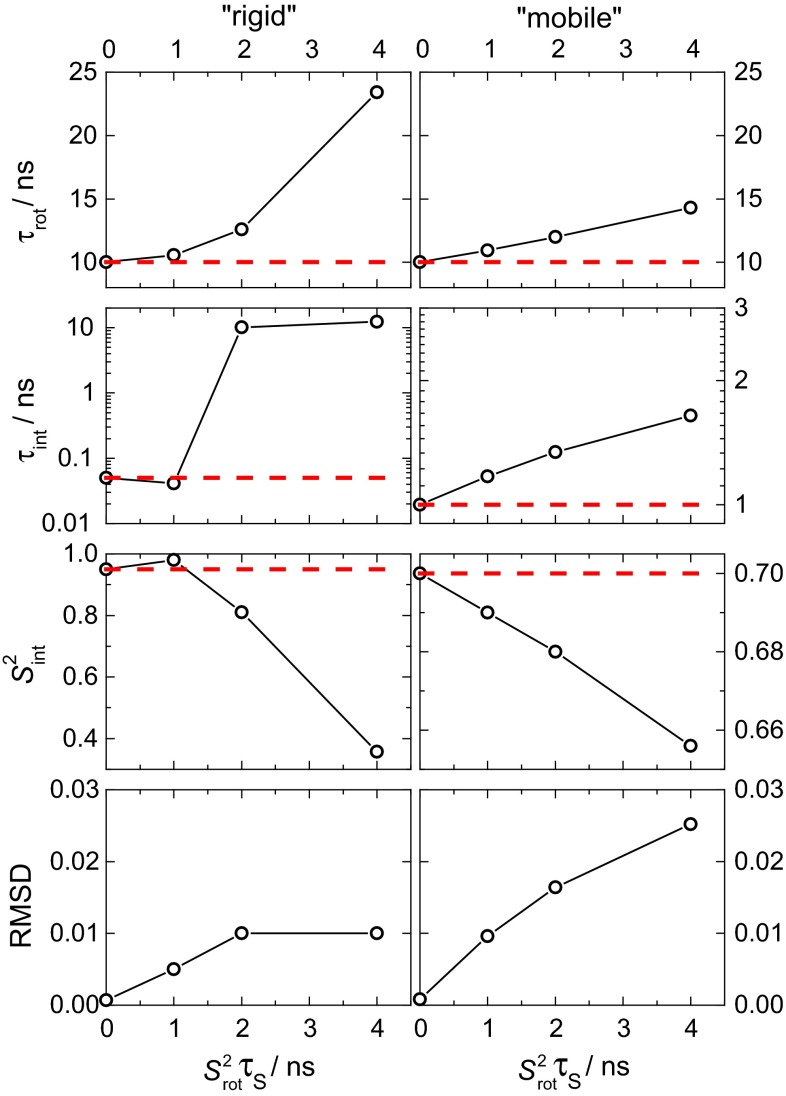
The values of the dynamic parameters τ_rot_, τ_int_ and $S_{\text{int}}^{2}$ as a function of the product $S_{\text{rot}}^{2}$ τ_S_ as obtained from fitting the simulated relaxation data assuming $S_{\text{rot}}^{2}$ = 0. The *red dotted lines* indicate the correct values of the parameters used in the simulations. The *two bottom plots* show the root mean square deviation (RMSD) of the fitting result

Figure 7 shows the values of the parameters τ_rot_, τ_int_ and $S_{\text{int}}^{2}$ obtained from fitting (with fixed $S_{\text{rot}}^{2}$ = 0) the simulated relaxation data generated with various $S_{\text{rot}}^{2}$ values (see above). See Fig. 1 for an illustration of the input (real) and the fitted (apparent) spectral densities for the “mobile” case with $S_{\text{rot}}^{2}$ = 0.02. It can be clearly seen that neglecting the “long tail” can lead to appreciably mismatching fitting parameters. Although the root mean square deviation of the fitting result increases with increasing $S_{\text{rot}}^{2} ,$ it, however, still remains quite small and reaches just a few percent, which corresponds to the typical experimental error of high-field NMR relaxation measurements. Again, this outcome demonstrates that the “long tail” *cannot* be seen in this type of analysis. If one aims at the precise NMR relaxation analysis of the protein dynamics in solution, one has either to combine high-field and low-field experiments, or to measure the concentration dependencies of the relaxation parameters in order to extrapolate them to zero concentration, where the “long tail” vanishes. This approach, however, would make the study obviously much more laborious.

It would be of interest to know the concentration from which on the inter-protein interactions can be assumed to be negligible. NMR is definitely not the best method for experimental detection of the “long tail” at very low concentrations since it suffers from the inherently low sensitivity in comparison with many other techniques. More sensitive methods for direct observation of inter-protein interactions are neutron and X-ray small-angle scattering (SAS) experiments (Roosen-Runge et al. 2011). Structure factors as obtained from SAS demonstrate that proteins cannot generally be assumed to act as non-interacting hard spheres. The inherent order of proteins in solutions is strongly affected by the strength of the repulsive electrostatic interactions, and consequently depends on the pH and ionic strength of the solvent (Velev et al. 1998) that influence the net charge and electrostatic screening, respectively.

Essentially, NMR and SAS methods observe the same phenomenon—long-range inter-protein electrostatic interactions. It is important, however, that the information provided by these methods is not similar but complementary: SAS and NMR report on spatial and orientational ordering of proteins, respectively, caused by electrostatic interactions. Thus, studies of protein solutions combining the capabilities of both methods will likely enable a deeper understanding of the nature of inter-protein interactions. At the moment we would like to attract reader’s attention to the studies (Stradner et al. 2004; Shukla et al. 2008; Heinen et al. 2012) in which SAS data were recorded in protein solutions in a wide concentration range. These results show that the spatial ordering (i.e. repulsive inter-protein electrostatic interactions) becomes negligible only at protein concentrations at about a few mg/ml. At such low concentrations, liquid-state NMR relaxation measurements are hardly possible without a cryoprobe.

## Conclusions

Mutual protein electrostatic steering is an essential effect in protein solution even at relatively low concentrations, which may appreciably affect the NMR relaxation rates, primarily *R*_2_. Neglecting it in the analysis of NMR relaxation data may lead to imprecise results, in particular at high concentrations. The parameters of the “long tail” of the rotational correlation function reveal different concentration dependencies: the intensity of the slow component (order parameter of the Brownian tumbling) expectedly increases with increasing concentration. However, its correlation time, i.e. the lifetime of the local anisotropy, decreases. This behavior can be understood when taking into account the shorter inter-protein distances at higher concentrations. Reduced inter-particle distances lead to an increased sensitivity to environmental fluctuations, which in turn decreases the life-time of the actual particle configuration. Literature data on X-ray and neutron small-angle scattering demonstrate the spatial ordering of proteins in solution, which complements the NMR results on the orientational ordering. As a more sensitive technique, small-angle scattering experiments demonstrate that protein spatial ordering is present at concentrations even below 1 mM, i.e. at the edge of the concentration range suitable for the NMR relaxation experiments.

## Electronic supplementary material

Explanation of the *R*_1ρ_ rescaling for a visual comparison with *R*_1_ data; discussion of a reliability of the FC R_1_ measurements at low fields; detailed experimental and fitting results for LYZ and BSA; discussion of the LYZ data fitting assuming no “long tail”; simulations of the high-field ^15^N NMR relaxation data. (PDF 573 kb)

## Appendix: Influence of the limited ramp time in FC-experiments on the shape of relaxation decay

In field-cycling experiments, the magnetic field is ramped down from the polarization field (here: ${{\omega_{0}^{{ ( {\text{pol)}}}} } \mathord{\left/ {\vphantom {{\omega_{0}^{{ ( {\text{pol)}}}} } {2\pi }}} \right. \kern-0pt} {2\pi }} = 20\,{\text{MHz}}$) towards the adjustable relaxation field, and is then ramped up again to the detection field (here: ${{\omega_{0}^{{ ( {\text{acq)}}}} } \mathord{\left/ {\vphantom {{\omega_{0}^{{ ( {\text{acq)}}}} } {2\pi }}} \right. \kern-0pt} {2\pi }} = 16\,{\text{MHz}}$). The ramp time of the FC relaxometry measurements was around 2 ms, and is thus comparable to the protein’s mean relaxation rate at low magnetic fields. The following section addresses the consequences for the multi-exponential relaxation decays as observed in this study.

In presence of a time dependent magnetic field, a *single*-*component* relaxation decay can be written as

12 $$ \frac{{I(t_{N} )}}{{I_{0} }} = \mathop {\lim }\limits_{{\Delta t \to 0}} \prod\limits_{i = 1}^{N} {\exp \left[ { - R_{1} \left( {\omega (t_{i} )} \right)\Delta t} \right]} ,\quad t_{N} = N\Delta t, $$

from which one can obtain a time-dependent mean relaxation rate $\bar{R}_{1} (t)$:

13 $$ \frac{I(t)}{{I_{0} }} = \exp \left[ { - \bar{R}_{1} (t)\;t} \right],\quad \bar{R}_{1} (t) = \frac{1}{t}\int_{0}^{t} {R_{1} \left( {\omega (t^{{\prime }} )} \right){\text{d}}t^{{\prime }} } . $$

Hence, the intensity after ramping down the magnetic field simply reads

14 $$ I^{{ ( {\text{rd)}}}} = \left( {I^{{ ( {\text{pol)}}}} - I^{{ ( {\text{rlx)}}}} } \right)\exp \left[ { - \bar{R}_{1}^{{ ( {\text{rd)}}}} \;\tau^{{ ( {\text{rd)}}}} } \right] + I^{{ ( {\text{rlx)}}}} , $$

where $I^{{ ( {\text{pol)}}}}$ and $I^{{ ( {\text{rlx)}}}}$ denote the equilibrium intensity at the polarization and relaxation field, respectively, $\tau^{{ ( {\text{rd)}}}}$ is the field-switching time, and $\bar{R}_{1}^{{ ( {\text{rd)}}}}$ is the mean relaxation rate during the magnetization ramp as defined by Eq. (13). After the magnetization ramp, the intensity is now subject to relaxation during the adjustable relaxation delay $t^{{ ( {\text{rlx)}}}} ,$ and takes place at the wanted relaxation field of frequency $\omega_{0}^{{ ( {\text{rlx)}}}} ,$

15 $$ I(t^{{ ( {\text{rlx)}}}} ) = \left( {I^{{ ( {\text{rd)}}}} - I^{{ ( {\text{rlx)}}}} } \right)\exp \left[ { - R_{1} (\omega_{0}^{{ ( {\text{rlx)}}}} )\;t} \right] + I^{{ ( {\text{rlx)}}}} . $$

Finally, ramping up the magnetic field for data acquisition during the field-switching time $\tau^{{ ( {\text{ru)}}}}$ causes the intensity to relax towards its new equilibrium magnetization $I^{{ ( {\text{acq)}}}} ,$ so that the signal finally detected reads

16 $$ \begin{aligned} I_{\text{acq}} (t^{{ ( {\text{rlx)}}}} ) & = \left( {I^{{ ( {\text{acq)}}}} - I(t^{{ ( {\text{rlx)}}}} )} \right)\left( {1 - \exp \left[ { - \bar{R}_{1}^{{ ( {\text{ru)}}}} \;\tau^{{ ( {\text{ru)}}}} } \right]} \right) + I(t^{{ ( {\text{rlx)}}}} ) \\ & = a\,\exp \left[ { - R_{1} (\omega_{0}^{{ ( {\text{rlx)}}}} )\;t^{{ ( {\text{rlx)}}}} } \right] + b, \\ \end{aligned} $$

where $\bar{R}_{1}^{{ ( {\text{ru)}}}}$ is the mean relaxation rate during increasing the magnetic field, and *a* and *b* are constants defined as

17 $$ a = \left( {I^{{ ( {\text{pol)}}}} - I^{{ ( {\text{rlx)}}}} } \right)\exp \left[ { - \left( {\bar{R}_{1}^{{ ( {\text{rd)}}}} \tau^{{ ( {\text{rd)}}}} + \bar{R}_{1}^{{ ( {\text{ru)}}}} \tau^{{ ( {\text{ru)}}}} } \right)} \right] $$

18 $$ b = I^{{ ( {\text{acq)}}}} \left( {1 - \exp \left[ { - \bar{R}_{1}^{{ ( {\text{ru)}}}} \;\tau } \right]} \right) + I^{{ ( {\text{rlx)}}}} \exp \left[ { - \bar{R}_{1}^{{ ( {\text{ru)}}}} \;\tau } \right]. $$

The above equations demonstrate that if the protein’s relaxation decay is single-exponential, the limited ramp time of the magnetic field would only influence the initial and final intensity of the decay observed, but not the relaxation rate.

In case of a multi-exponential decay, in turn, the amplitude *a* and the equilibrium value *b* may be different for the individual components of the decay. A change in the offset *b* from one proton site to another is of no relevance for correctly estimating the mean relaxation rate $\left\langle {R_{1} (\omega_{0}^{{ ( {\text{rlx)}}}} )} \right\rangle = \sum\nolimits_{\text{i}} {{\kern 1pt} a_{\text{i}} \,R_{{ 1 , {\text{i}}}} (\omega_{0}^{{ ( {\text{rlx)}}}} )} ,$ as the plateau value of the relaxation decay is independently fitted, yet modified amplitudes *a*_i_ do affect a correct estimate of $\left\langle {R_{1} (\omega_{0}^{{ ( {\text{rlx)}}}} )} \right\rangle .$ The faster the relaxation during the magnetization ramp, the smaller is the contribution of this component to the experimentally observed decay. This issue is only of relevance at measurements at low relaxation fields, as only those magnetization ramps involve fast relaxation occurring at low magnetic fields. The potential decrease of the amplitude of quickly relaxing components may hence cause an underestimation of the relaxation rate at low frequencies.

The presence of BSA oligomers in solution causes an additional spread of the relaxation rates, rendering the issue of multi-component decays problematic at low relaxation fields. Thus, measurements in BSA solutions at relaxation fields below 3 MHz ^1^H Larmor frequency were not taken into account, also due to the reasons discussed in ESM, Fig. S2.

The mean relaxation rate of LYZ, however, could be safely estimated even at relaxation fields as low as 0.1 MHz: fitting the relaxation decays provides stable results (Fig. S2) with a distribution width of relaxation times reaching a plateau value at low frequencies (Fig. 4), thus indicating that the *shape* of the FC-NMR relaxation decay is the same at different magnetic fields. At low magnetic fields, where relaxation is fast as compared to spin exchange, the distribution width σ reflects the intrinsic spread of relaxation times of the protein protons, and hence has to be independent of the actual magnetic field applied. If, however, relaxation during the magnetization ramps reduced the contribution of quickly relaxing proton sites to the overall signal, the experimentally observed distribution of relaxation times would become narrower at low magnetic fields. Such a trend cannot be seen in the distribution width (Fig. 4), thus indicating that the magnetization ramps have no significant effect on the mean relaxation rate in LYZ solutions.
