# Supplementary material for: The “long tail” of the protein tumbling correlation function: observation by 1H NMR relaxometry in a wide frequency and concentration range
Source: J Biomol NMR. 2015 Nov 18;63(4):403–15. doi: 10.1007/s10858-015-0001-1 (PMC4662726; doi:10.1007/s10858-015-0001-1)
Supplement: Supplementary file 1 — Explanation of the R 1ρ rescaling for a visual comparison with R 1 data; discussion of a reliability of the FC R1 measurements at low fields; detailed experimental and fitting results for LYZ and BSA; discussion of the LYZ data fitting assuming no “long tail”; simulations of the high-field 15N NMR relaxation data. (PDF 573 kb) [file 10858_2015_1_MOESM1_ESM.pdf]

Electronic Supporting Material

The "long tail" of the protein tumbling correlation function: observation by ^{1}H NMR relaxometry in a wide frequency and concentration range.

Matthias Roos[1], Marius Hofmann[2], Susanne Link[1], Maria Ott[1], Jochen Balbach[1], Ernst Rössler[2], Kay Saalwächter,[1]* Alexey Krushelnitsky[1]*

[1] Martin-Luther-Universität Halle-Wittenberg, Germany;

[2] University of Bayreuth, Germany

Rescaling of $R_{1\rho}$ data for visual comparison with R_1 data

For visual comparison only, $R_{1\rho}$ was rescaled by a factor of 10/3 to be in accordance with the absolute values of the R_1 data. This scaling factor directly follows from the approximation $J(\omega_0) \approx J(2\omega_0)$, providing

$$R_1 \approx \frac{10}{3} K_{\mathrm{HH}} J(2\omega_0) \tag{S1}$$

$$R_{1\rho} \approx \frac{K_{\mathrm{HH}}}{3}\left[3J(2\omega_{\mathrm{SL}}) + 7J(2\omega_0)\right] \tag{S2}$$

$$R_2 \approx \frac{K_{\mathrm{HH}}}{3}\left[3J(0) + 7J(2\omega_0)\right] \; . \tag{S3}$$

Making use of $J(2\omega_{\mathrm{SL}}) >> J(2\omega_0)$ in the case of the $R_{1\rho}$ measurements, in which $\omega_{\mathrm{SL}} / 2\pi \le 40\,\mathrm{kHz}$ and $\omega_0 / 2\pi = 400\,\mathrm{MHz}$, it follows that

$$R_1 \approx \frac{10}{3} K_{\mathrm{HH}} J(2\omega_0) \tag{S4}$$

$$\frac{10}{3} R_{1\rho} \approx \frac{10}{3} K_{\mathrm{HH}} J(2\omega_{\mathrm{SL}}) \; . \tag{S5}$$

Thus, the two quantities that map out $J(\omega)$ can be compared visually in the same plot.

In contrast, our R_2 measurements were performed at a Larmor frequency of 20 MHz, which does not allow for safely neglecting the contribution of $J(2\omega_0)$ to R_2. A correct rescaling of

R_2 thus requires knowledge on both $J(\omega_0)$ and $J(2\omega_0)$, which is impossible without further data analysis. Of course, data fitting was performed using the exact equations for R_1, $R_{1\rho}$ and R_2.

Reliability of the FC-NMR R_1 measurements at low fields

The shortest relaxation delay in our field-cycling measurements was 0.7 ms ("dead time"). In the presence of a distribution of R_1s, the initial slope of the field-cycling relaxation decay may thus not reflect the true initial slope at t=0, as illustrated by Fig. S1.

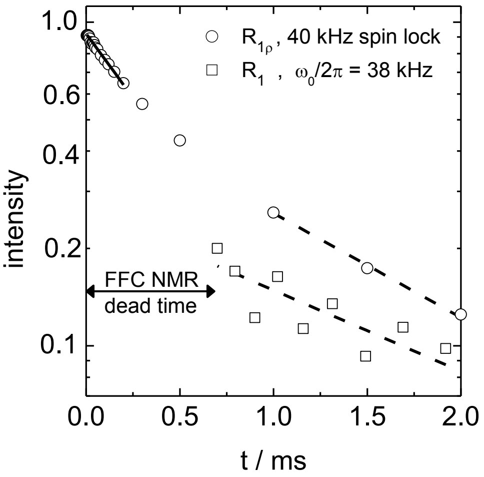

Figure S1. BSA (c=213 mg/ml, T=20 ºC) $R_{1\rho}$ and R_1 relaxation decays after the water component subtraction. As seen from the $R_{1\rho}$ decay, the experimentally observed relaxation rate (solid and dashed line) may significantly change during the dead time of the FC NMR measurements. This issue is of relevance as soon as the relaxation time of the fast relaxing protein component becomes comparable to the dead time.

The relaxation decays were only considered as being reliable if a bi-exponential fit to the data provided - within the fitting uncertainty - the same initial slope as the fitting result based upon a log-normal distribution, see Fig. S2. In the case of bi-exponential analysis, the relaxation decay was fitted according to the equation

$$A(t) = P_a \exp(-R_a t) + P_b \exp(-R_b t) \quad , \qquad \text{(S6)}$$

where $P_{a,b}$ and $R_{a,b}$ are the intensities and relaxation rates of the components a and b. Then, the mean relaxation rate was defined as

$$\langle R \rangle = \frac{P_a R_a + P_b R_b}{P_a + P_b} \quad . \qquad \text{(S7)}$$

If the two evaluation methods gave different results, the distribution of R_1s within the undetected part of the decay (initial 0.7 ms), and thus the estimate of $<R_1>$, was considered uncertain. This situation occurs in the case of BSA at low relaxation fields, potentially due to the presence of oligomers, as these cause increased relaxation rates and an increased curvature of the relaxation decays.

For the sake of clarity in notation, the brackets of $<R_1>$ will henceforth be skipped, as is also the case in the main paper.

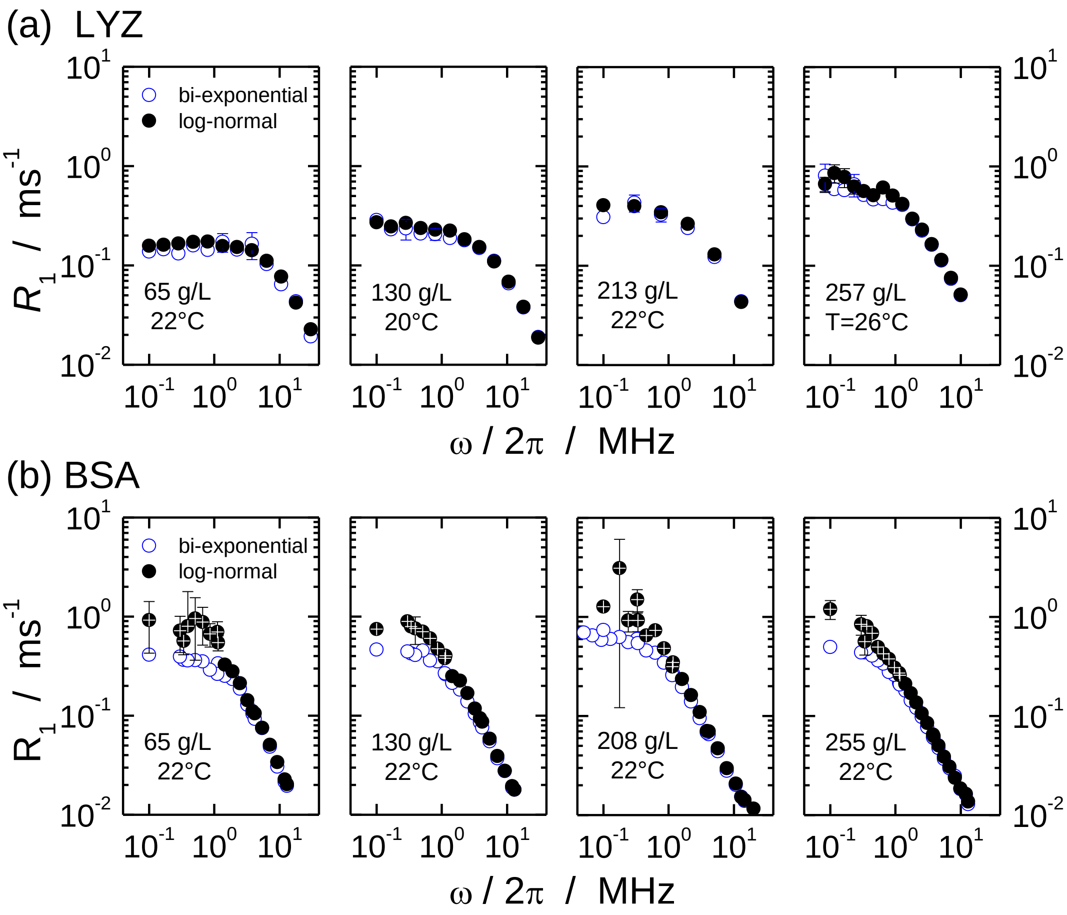

Figure S2. Relaxation rates of (a) LYZ and (b) BSA as provided by bi-exponential (open symbols) and log-normal-distribution fitting to the raw data. If not indicated by error bars, the fitting error was of the same order of magnitude as or smaller than the symbol size. Data points marked with a cross were not used for further data analysis.

An additional argument supporting the accuracy of the FC-NMR measurements at low field in LYZ solution is provided by the comparison of R_1 with $R_{1\rho}$ measurements, as the latter provide a safe, dead-time free estimate of the relaxation rate at low frequencies in absence of any relevant delays during the pulse sequence. At 65 mg/ml and 130 mg/ml protein concentration, the FC-NMR measurements provide a plateau value of R_1 that at all temperatures matches the corresponding value of 10/3 $R_{1\rho}$ (see Fig. S3). The same holds for the samples of higher protein concentrations (213 mg/ml, 257 mg/ml) at temperatures above 15°C. At lower temperatures of the highly concentrated samples, however, no plateau of R_1 is

observed, which we address to the increased relevance of the slow component of rotational diffusion. However, to be safe with respect to the influence of potentially biased data points in highly concentrated LYZ solutions, we excluded FC-NMR values of a mean relaxation rate above 0.5 ms^{-1} (at and below this value, R_1 reaches a plateau, in agreement with $R_{1\rho}$) from data fitting, and observed very similar results compared to the fit of all data without exceptions, see Table S1.

Experimental and fitting results

(i) LYZ

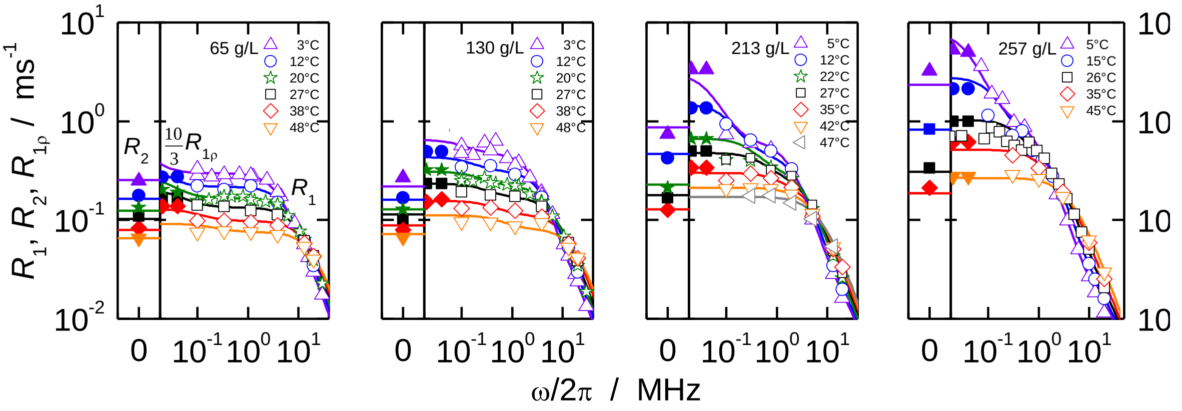

Figure S3. Dispersion profiles of LYZ at several temperatures. The shown data points include the full FC-R_1 data set, a part of it being shown in Fig. 5 of the main paper. $10/3R_{1\rho}$ data points are shown as solid symbols. Additionally, we here include data points for R_1 measured with a saturation recovery sequence on a Bruker minispec mq20 at the resonance frequency 20 MHz. In order not to overload the figure, error bars were skipped, the measuring uncertainty of the relaxation rates is comparable to the symbol size. A part of these data plotted as a function of temperature is shown in Fig. S5a.

Table S1. Fit result of LYZ. The values in row A are the best-fit results including all data points measured. For row B potentially biased data points ($\langle R_1 \rangle > 0.5$ ms^{-1}) were excluded (see explanation above).

		Brownian rotation		"Long tail" of rot. diffusion			Internal dynamics			
	c g/L	τ_{rot} ns	E_{rot} kJ/mol	S^2_{rot}	τ_S ns	E_S kJ/mol	τ_{int} ns	S^2_{int}	E_{int} kJ/mol	$K^{(av)}_{HH}$ 10^9 s²
**A**	**65**	**10.5±0.2**	**25±1**	**≤0.003**	**≥3000**	**54±2**	**2.3±0.1**	**0.72 ± 0.01**	**6±3**	**6.1 ±0.1**
	**130**	**14.6±0.2**	**31±1**	**0.014±0.002**	**410±40**	**29±1**				
	**213**	**23.9±0.3**	**27±1**	**0.09±0.01**	**330±10**	**73±1**				
	**257**	**39.9±0.6**	**35±2**	**0.23±0.01**	**380±10**	**76±1**				
B	65	10.5±0.1	24±1	≤0.003	≥3000	53±2	2.2±0.1	0.73 ± 0.01	9±1	6.1 ±0.1
	130	13.9±0.2	29±1	0.016±0.001	390±30	33±1				
	213	23.3±0.4	30±1	0.13±0.01	240±10	65±1				
	257	38.1±0.7	36±1	0.23±0.01	410±10	73±1				

(ii) BSA

Commercially available BSA contains a significant fraction of oligomers in solution. If these are neglected upon fitting the data, the procedure will give a mean rotational correlation time that incorporates an uncertain weighting of monomers *vs*. oligomers. However, since $\tau_S >> \tau_{rot}$ a spread of the rotational correlation does not challenge the sensitivity of the fit to the slow component: R_2 and $R_{1\rho}$ values are most sensitive to the latter, whereas relaxation at higher Larmor frequencies serves to fix τ_{rot}.

Taking into account the oligomer fraction increases the number of fitting parameters, but provides more reasonable results. To reduce the complexity of the fitting model (otherwise, the fitting is too uncertain and ambiguous), we assumed the oligomer fraction P_M to stay the same over the entire temperature range ($4°C \leq T \leq 30°C$), and fixed the rotational activation energy of oligomers and monomers at all concentrations to 20 kJ/mol, that is, the activation energy of water flow that governs the protein rotational motion (in the case of LYZ and αB-crystallin (Roos et al. 2015) we obtained similar values of E_{rot}). We divided the rotational correlation time into two components, τ_{rot} and τ_{rot2}, that correspond to the monomer and oligomer fractions, respectively. In practice, the second term of $J(\omega)$, see Eq. (7) of the main article, was replaced by

$$S_{int}^2\left(1-S_{rot}^2\right)\left[\frac{P_M\tau_{rot}}{1+\left(\omega\tau_{rot}\right)^2}+\frac{(1-P_M)\tau_{rot2}}{1+\left(\omega\tau_{rot2}\right)^2}\right]. \qquad \text{(S8)}$$

Figs. S4 and S5 displays the corresponding fitting results. According to the data analysis, oligomers comprise more than half of the protein mass. This outcome is in accordance with our size-exclusion chromatography results obtained for dilute BSA solutions (cf. Materials and Methods), and further agrees well with the findings of ref. (Atmeh et al. 2007). Table S2 presents the fitting results for the two options of the data analysis (single τ_{rot} and τ_{rot}/τ_{rot2} components of the overall tumbling correlation function).

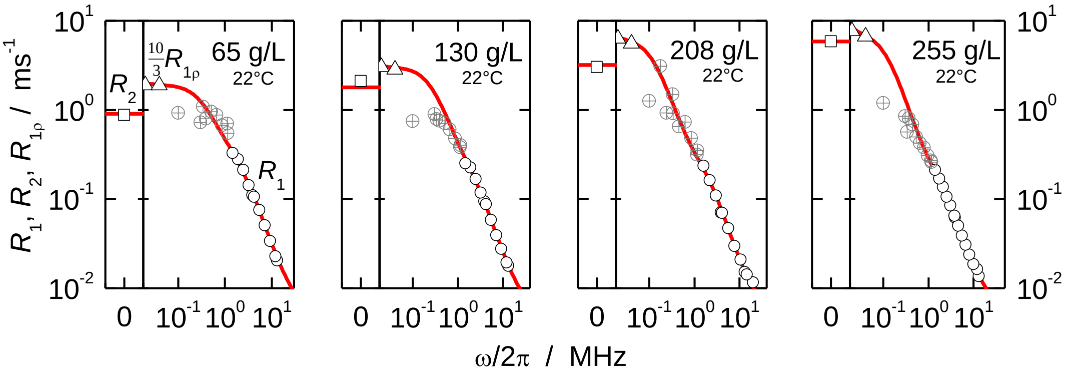

Figure S4. Dispersion profiles of the BSA relaxation data (the same as shown in Fig. 5b of the main paper). Fitting curves (solid red lines) correspond to the analysis that takes into account oligomers (Eq. S8). Crossed symbols are uncertain data points that were not included in data fitting (see explanation in the text). Other BSA data are shown as function of temperature in Fig. S5b.

Table S2. Fitting results of BSA, excluding uncertain data points. Row A is from fits excluding oligomers, in which case τ_{rot} represents an average over monomers and oligomers. Row B is the best fit result including oligomers, where "M" and "O" denote monomers and oligomers, respectively. The oligomer fractions P_M (see Eq. S8) are 0.45±0.05, 0.60±0.05, 0.60±0.05 and 0.70±0.08 for the concentrations 65, 130, 208 and 255 g/L, respectively.

		Brownian rotation		"Long tail" of rot. diffusion			Internal dynamics			
	c g/L	τ_{rot} ns	E_{rot} kJ/mol	S^2_{rot}	τ_S μs	E_S kJ/mol	τ_{int} ns	S^2_{int}	E_{int} kJ/mol	$K^{(av)}_{HH}$ 10^9 s²
A	65	107±1	17±1	≤0.002	≥20	40±3	2.0±0.1	0.79±0.01	0.2±0.1	7.8±0.1
	130	158±1	18±1	0.006±0.001	23±4	39±2				
	208	203±3	23±1	0.10±0.01	3±1	45±1				
	255	263±4	19±1	0.12±0.01	6±1	45±2				
**B**	**65**	M: **36±2**<br>O: **260±20**	**20** (*)	**≤0.002**	**≥30**	**29±5**	**1.4±0.1**	**0.74±0.01**	**5±4**	**6.0±0.1**
	**130**	M: **35±2**<br>O: **330±20**	**20** (*)	**0.006±0.002**	**29±7**	**37±2**				
	**208**	M: **45±2**<br>O: **730±30**	**20** (*)	**0.02±0.01**	**15±2**	**62±2**				
	**255**	M: **42±2**<br>O: **760±23**	**20** (*)	**0.05±0.01**	**15±2**	**52±2**				

(*) To stabilize these fits, the activation energy of τ_{rot} was fixed to 20 kJ/mol at all concentrations.

(iii) Relaxation rates as a function of temperature

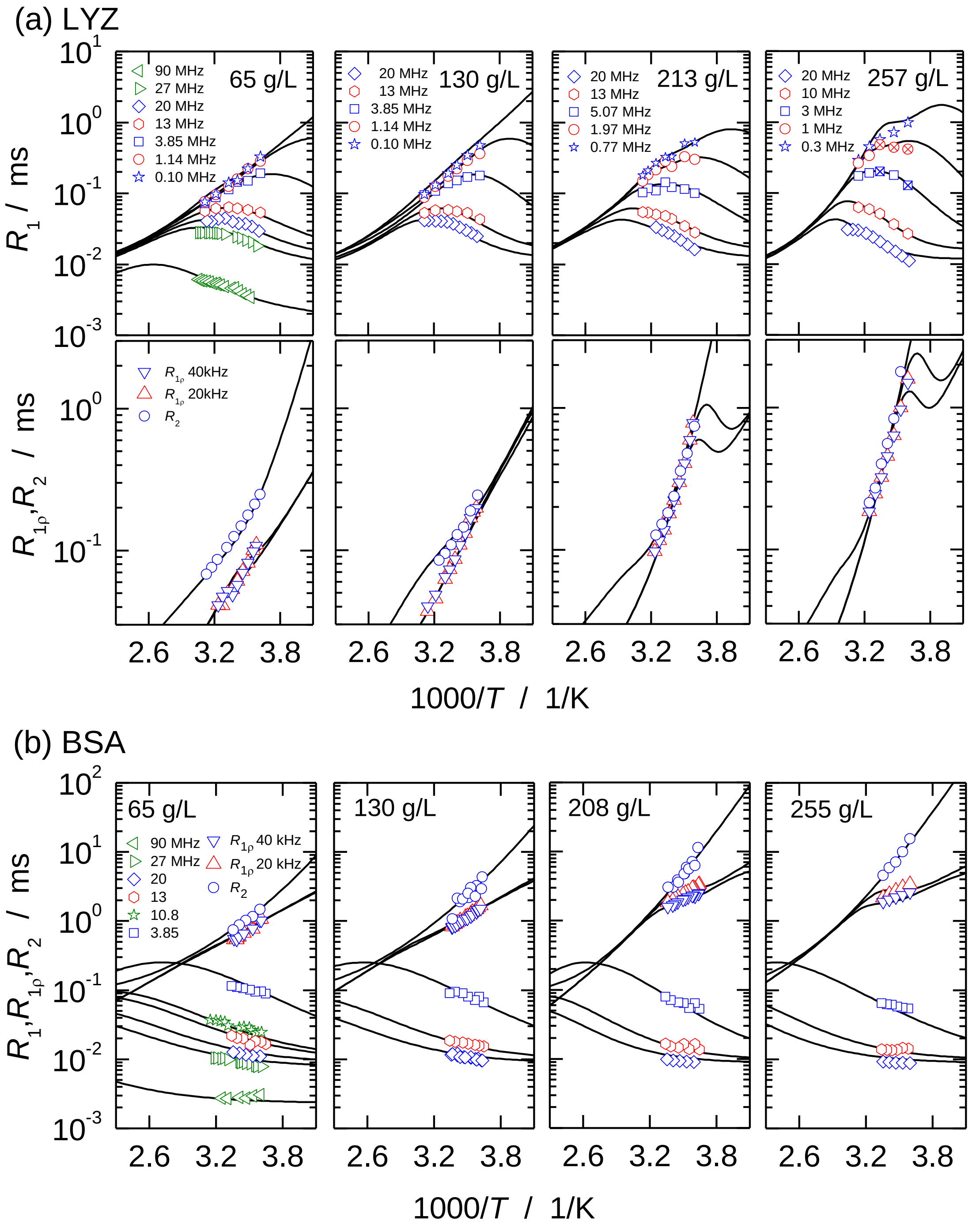

Figure S5.Temperature dependencies of R_1, $R_{1\rho}$ and R_2 relaxation times together with the best fit results (solid lines). R_1 values at 90, 27 and 10.8 MHz were taken from ref. (Krushelnisky 2006). Two symbols marked with a cross (LYZ 257 g/L) were interpolated from measurements at different frequencies but at the same temperature, and are only shown for visual presentation, but were not included in the fitting.

Fitting LYZ data assuming no "long tail" component

The necessity of inclusion of the "long tail" in the fitting model was justified in our previous papers, see the Introduction. Here, by the example of LYZ data, we demonstrate once more that neglecting the "long tail" provides a systematic inconsistency between experimental points and fitting curves. Fig. S6 presents the LYZ relaxation data in frequency and temperature domains along with the fitting curves assuming $S^2_{\text{rot}} = 0$.

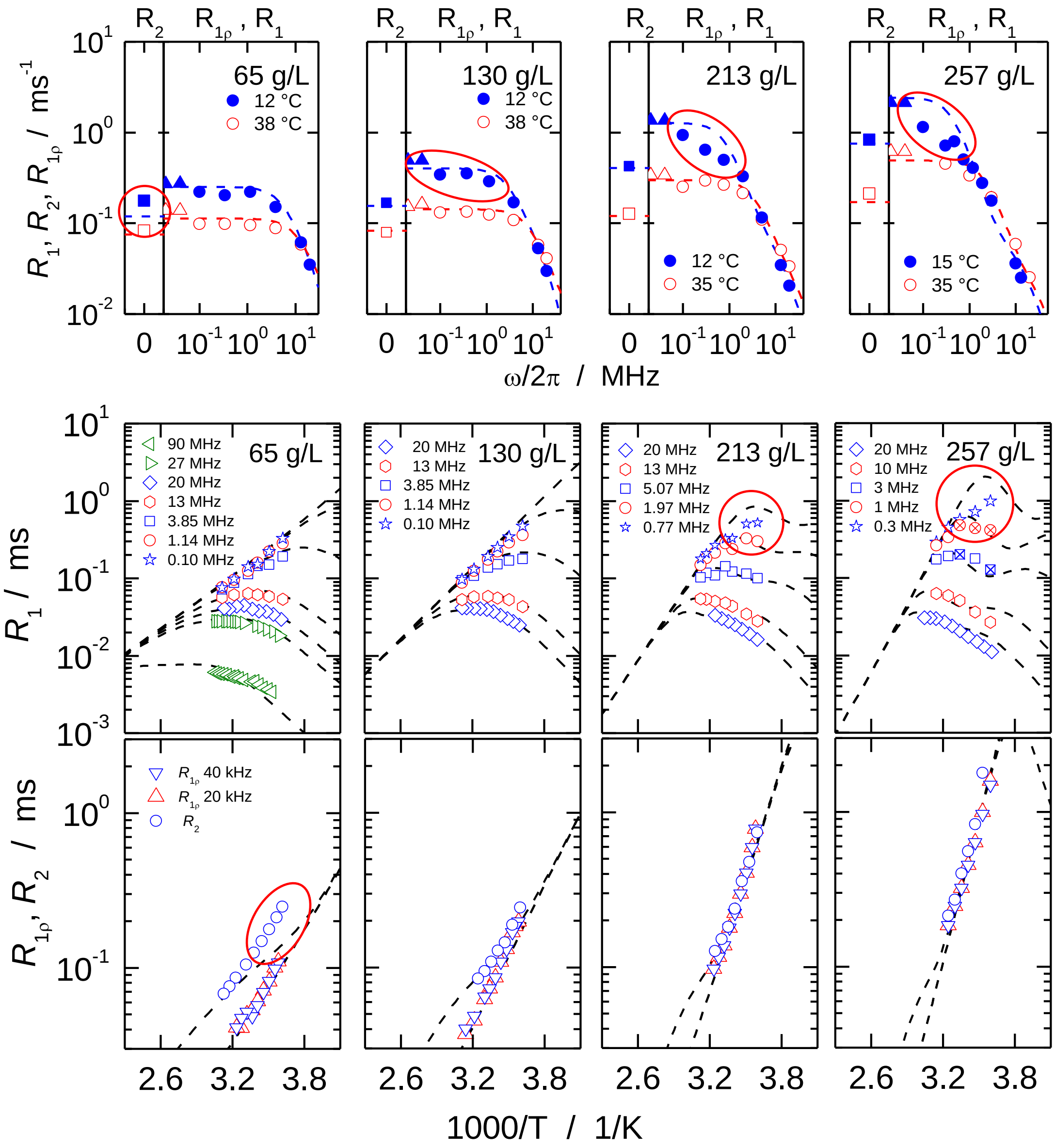

Figure S6. Experimental data and the fitting curves for LYZ solutions. Fitting was performed assuming $S^2_{\text{rot}} = 0$. The regions of systematic inconsistency between the experimental and fitting relaxation rates values are indicated by red circles/ovals.

As seen in that graph, at low concentration R_2 values cannot be reasonably fitted. The correlation time τ_S at this concentration is large, and thus it affects only R_2; all other

relaxation data can be described by the simplest model quite well. Upon increasing concentration, τ_S becomes shorter (see the discussion of this phenomenon in the main paper), and thus the region of the fitting inconsistency shifts towards FC R_1's in the frequency range 0.5 - 1 MHz. The higher the concentration, the larger the order parameter S^2_{rot} and, hence, the larger the fitting discrepancy, which is seen in Fig. S6. Finally, we note that the root mean square deviation of the fitting (see Eq. 11 of the main paper) with and without the "long tail" is 0.12 and 0.2, respectively.

Simulation of high-field ^{15}N NMR relaxation data.

The ^{15}N relaxation parameters were simulated according to the standard formulae for the heteronuclear dipole-dipole and CSA relaxation mechanisms with a typical values for the N–H pair in the protein backbone (see e.g. Daragan and Mayo 1997). Below we show the simulated R_1, R_2 and NOE's for three frequencies as a function of the product $S^2_{rot}\,\tau_S$ for the "rigid" and "mobile" residues:

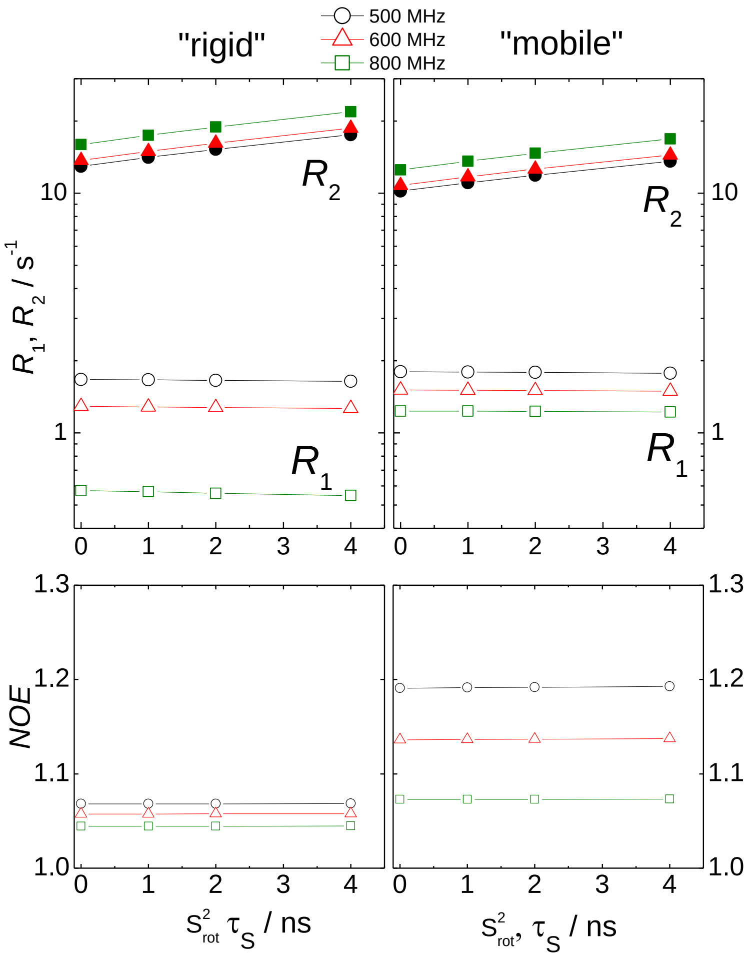

Figure S7. Simulated R_1, R_2 and NOE's as a function of the product $S^2_{rot}\tau_S$ for "rigid" and "mobile" residues.

References

Atmeh RF, Arafa IM, Al-Khateeb M (2007) Albumin aggregates: hydrodynamic shape and physico-chemical properties. Jordan J Chem 2:169-182.

Daragan VA, Mayo KH (1997) Motional model analyses of protein and peptide dynamics using C-13 and N-15 NMR relaxation. Prog NMR Spect 31:63-105.

Krushelnitsky A (2006) Intermolecular electrostatic interactions and Brownian tumbling in protein solutions. Phys Chem Chem Phys 8:2117–2128.

Roos M, Link S, Balbach J, Krushelnitsky A, Saalwächter K (2015) NMR-detected Brownian dynamics of αB-crystallin over a wide range of concentrations. Biophys J 108:98-106.
